# Clinical utility of jugular venous blood gas biomarkers for assessing cranial brain injury severity and predicting ICU stay duration: A biochemical and predictive modeling approach

**DOI:** 10.5937/jomb0-59108

**Published:** 2025-11-05

**Authors:** Bin Qi, Wenjie Du, He Zhang, Yuhui Jiang, Ming Zhuo, Haixia Xu

**Affiliations:** 1 Bozhou People's Hospital, Department of Intensive Care Medicine, Economic Development Zone, Bozhou, China; 2 Bozhou People's Hospital, Department of Cardiovascular Medicine, Economic Development Zone, Bozhou, China

**Keywords:** jugular venous blood gas, cranial brain injury, cerebral oxygenation, biochemical markers, ICU prediction, machine learning, gasna krv u jugularnoj veni, traumatska povreda mozga, cerebralna oksigenacija, biohemijski markeri, predviđanje na intenzivnoj nezi, mašinsko učenje

## Abstract

**Background:**

Biochemical monitoring of cerebral oxygen metabolism is essential in the management of cranial brain injury (CBI). Jugular venous blood gas (JVBG) analysis provides real-time data reflecting cerebral biochemical states, offering a valuable window into brain oxygenation and metabolic demands. This study aimed to evaluate JVBG biochemical markers as indicators of CBI severity and as predictors of prolonged intensive care unit (ICU) stay, emphasizing their integration into machine learning models for clinical utility.

**Methods:**

A retrospective analysis was conducted on 100 ICU-admitted CBI patients. Serial measurements of JVBG parameters-jugular venous oxygen saturation (SjvO2), partial pressure of oxygen (PjO2), arteriovenous oxygen difference (AVDO2), and oxygen content difference (AVDL)-were performed over five days. Spearman correlation and random forest algorithms were employed to assess relationships between JVBG biomarkers, clinical severity (via Glasgow Coma Scale), and ICU duration.

**Results:**

Patients with more severe CBI exhibited significantly reduced SjvO2 and PjO2, and elevated AVDO and AVDL (p &lt; 0.001). Strong correlations were found between JVBG markers and clinical severity scores. A multivariable prediction model incorporating age, SaO2, and PaCO2 at one day post-admission yielded excellent predictive performance for prolonged ICU stay (AUC = 0.974; sensitivity = 100%; specificity = 94.8%).

**Conclusions:**

JVBG biomarkers serve as clinically informative biochemical indicators for assessing CBI severity and forecasting ICU duration. Their integration into predictive algorithms may enhance precision in neurocritical care and support biochemical decision-making in intensive care settings.

## Abstract (sr)

**Uvod:**

Biohemijsko praćenje metabolizma cerebralnog kiseonika je neophodno u lečenju traumatske povrede mozga (TPM). Analiza gasova u jugularnoj venskoj krvi (JVBG) pruža podatke u realnom vremenu koji odražavaju cerebralna biohemijska stanja, nudeći dragocen uvid u oksigenaciju mozga i metaboličke potrebe. Cilj ove studije je bio da se proceni biohemijski markeri JVBG kao indikatori težine TPM i kao prediktori produženog boravka na odeljenju intenzivne nege (JIN), sa naglaskom na njihovoj integraciji u modele mašinskog učenja za kliničku primenljivost.

**Metode:**

Retrospektivna analiza je sprovedena na 100 pacijenata sa traumatskom traumom mozga primljenih na intenzivnu negu. Serijska merenja parametara jugularnog venskog grla (JVBG) - zasićenosti kiseonikom jugularne vene (SjvO2), parcijalnog pritiska kiseonika (PjO2), arteriovenske kiseoničke razlike (AVDO2) i razlike sadržaja kiseonika (AVDL) - vršena su tokom pet dana. Spirmanova korelacija i algoritmi slučajne šume korišćeni su za procenu veza između biomarkera jugularnog venskog grla, kliničke težine (preko Glazgove skale kome) i trajanja intenzivne nege.

**Rezultati:**

Pacijenti sa težom traumatičkom povredom mozga pokazali su značajno smanjene SjvO2 i PjO2, i povišene AVDO2 i AVDL (p &lt; 0,001). Pronađene su jake korelacije između markera JVBG i kliničkih ocena težine bolesti. Multi-varijantni model predikcije koji uključuje starost, SaO2 i PaCO2 jedan dan nakon prijema dao je odlične prediktivne performanse za produženi boravak na intenzivnoj nezi (AUC = 0,974; osetljivost = 100%; specifičnost = 94,8%).

**Zaključak:**

Biomarkeri JVBG služe kao klinički informativni biohemijski indikatori za procenu težine traumatske povrede mozga i predviđanje trajanja intenzivne nege. Njihova integracija u prediktivne algoritme može poboljšati preciznost u neurokritičnoj nezi i podržati biohemijsko donošenje odluka u ustanovama intenzivne nege.

## Introduction

Cranial brain injury (CBI) remains a considerable public health concern, with significant associated morbidity and mortality rates [1] [2] [3]. In addition to high mortality, CBI has a profound and lasting impact on the quality of life of patients, making it one of the leading causes of long-term disability. Survivors of moderate-to-severe CBI may suffer from chronic neurological deficits even after recovery, including sequelae such as motor disorders (e.g., hemiplegia), cognitive decline (e.g., memory loss, executive dysfunction), and communication disorders (e.g., aphasia). CBI can lead to immediate and long-term physical, cognitive, emotional, and functional impairments, resulting in substantial economic and societal burdens on affected individuals, their families, and healthcare systems globally [4] [5] [6]. Moreover, these deficits not only limit daily activities (e.g., self-care, mobility), but also contribute to psychological disorders - post-traumatic stress disorder (PTSD) or major depression - further reducing quality of life. Therefore, timely treatment and condition assessment of CBI patients is the key link in clinical management, which is of great significance for preventing long-term complications, improving prognosis and promoting the overall rehabilitation of patients. By closely monitoring the changes of patients' condition and taking targeted intervention measures, the physical and psychological burden caused by CBI can be effectively reduced and the quality of life of patients can be improved. The severity of CBI was determined by the Glasgow Coma Scale (GCS) score, with severe CBI defined as a GCS score of 8 or less [7] [8]. Patients with severe CBI often require admission to intensive care units (ICU) for close neurological monitoring, resuscitation, and management of potential complications such as hypoxia, hypotension, or elevated intracranial pressure [9] [10]. Jugular venous blood gas (JVBG) analysis has emerged as a promising tool for the assessment of CBI severity and prognostication of patient outcomes [11] [12].

Jugular venous blood provides a direct sample of blood drained from the brain, reflecting cerebral metabolism and oxygenation, and was increasingly recognized as a valuable source of physiological information in patients with CBI [13] [14] [15]. The constituents of JVBG, including oxygen tension (PvO_2_), carbon dioxide tension (PvCO_2_), and the arterio-jugular venous oxygen content difference (AVDO_2_), have been studied for their potential correlation with the severity and outcome of CBI [16] [17] [18]. Several studies have reported that abnormal JVBG parameters were associated with poor outcomes and increased mortality in patients with CBI [19] [20] [21].

Moreover, the predictive role of JVBG parameters in determining the length of ICU stay in CBI patients was an area of growing investigation [22]. Prolonged ICU stay not only imposes a considerable financial burden on healthcare systems but was also reflective of the complexity of patient management, severity of illness, and risk of adverse events [23]. Therefore, understanding the relationship between JVBG parameters, CBI severity, and length of ICU stay has the potential to impact clinical decision-making, resource allocation, and prognostication in patients with severe CBI. Given the evolving landscape of CBI management, there was a critical need to comprehensively evaluate the correlation between JVBG analysis, CBI severity, and its predictive role in the length of ICU stay.

This study aimed to systematically explore the correlation between JVBG analysis and the severity of CBI, while also investigating the potential of JVBG parameters as predictive markers for the length of ICU stay. By elucidating the relationship between JVBG parameters and CBI outcomes, we seek to contribute to the expanding knowledge base in CBI prognostication and potentially inform the development of targeted therapeutic strategies.

## Materials and methods

### Inclusion and exclusion criteria

Inclusion Criteria: Adult patients aged 18 years and above; Patients diagnosis of CBI; Admission to the ICU for management of CBI.

Exclusion Criteria: Patients below the age of 18; Patients with incomplete baseline data or missing parameters required for analysis; Patients with preexisting conditions or comorbidities that may significantly impact JVBG analysis parameters, such as severe cardiopulmonary disease, chronic obstructive pulmonary disease, or severe anemia; Patients with incomplete JVBG analysis data at the specified time points post-admission; Patients who did not receive ICU management for CBI.

**Figure 1:**
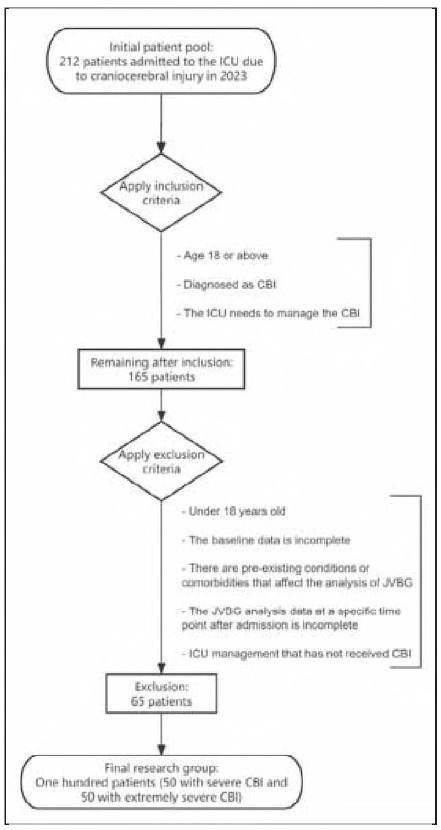
Patient Inclusion Exclusion Flowchart.

The inclusion and exclusion process is shown in Figure 1.

### Study population

This study conducted a retrospective analysis of clinical data from 100 patients with CBI admitted to the ICU of the Bozhou People's Hospital in 2023. The patients were divided into two groups: 50 cases with severe CBI and 50 cases with extremely severe CBI.

### Grouping criteria

The GCS was a neurological scale that assesses a patient's level of consciousness. The GCS ranges from 3 to 15, with 15 being the best score representing normal consciousness and 3 being the worst score indicating deep unconsciousness. Below was a breakdown of the GCS scores and their associated levels of consciousness: GCS 13-15: Mild brain injury or altered mental status; GCS 9-12: Moderate brain injury; GCS 3-8: Severe brain injury. In this study, patients with a GCS score of 3-5 were categorized as having extremely severe CBI, while those with a GCS score of 6-8 were categorized as having severe CBI.

### Jugular venous blood gas analysis

The blood sampling method for the jugular venous bulb involved retrograde blood collection via the jugular vein. With bedside ultrasound assistance, the tip of the catheter was advanced to the position of the jugular venous bulb, with the catheter inserted approximately 8-12 cm into the jugular vein to collect blood from the mastoid vein, at a blood withdrawal rate of <2 mL/min. After sealing the catheter with heparin saline solution (5 μ/mL), a heparin cap was placed for blood sampling through this catheter. No dedicated indwelling catheter was left in place.

### Glasgow Outcome Scale (GOS)

The Glasgow Outcome Scale (GOS) was used to measure the overall outcome and recovery of patients who have suffered a brain injury. It categorizes patients into one of five outcomes. Dead: Patient has died as a result of the brain injury; Vegetative State: Patient was unaware of themselves and their surroundings, but has a sleep-wake cycle; Severe Disability: Patient was conscious but disabled and dependent on others for daily support; Moderate Disability: Patient was independent in daily life but may have some physical or mental deficits; Good Recovery: Patient has made a complete recovery or has minor physical or mental deficits.

### Data collection

Baseline data including gender, presence of cerebral trauma, cerebral hernia, intracranial hemorrhage, cerebral infarction, cerebral artery stenosis, death, GOS, GCS, age, and Acute Physiology and Chronic Health Evaluation (APACHE) score were collected from medical records. JVBG analysis parameters including jugular venous oxygen saturation (SjvO_2_), jugular venous partial pressure of oxygen (PjO_2_), arteriovenous oxygen difference (AVDO_2_), arteriovenous oxygen content difference (AVDL), blood lactate (BlA), hemoglobin, arterial oxygen saturation (SaO_2_), partial pressure of oxygen in arterial blood (PaO_2_), and partial pressure of carbon dioxide in arterial blood (PaCO_2_) were measured at one, two, three, four, and five days after admission.

### Statistical analysis

Data were analyzed using SPSS 25.0 (SPSS Inc., Chicago, IL, USA). Categorical variables were expressed as n (%) and compared using the chi-square test or Fisher's exact test, as appropriate. Continuous variables were analyzed using the t-test or Wilcoxon rank-sum test based on distribution. Spearman correlation was used to assess relationships between variables. A random forest model was developed to predict ICU stays longer than 14 days. We selected variables for the model based on their clinical relevance and univariate analysis results. Internal validation was performed using 5-fold cross - validation to ensure the stability and accuracy of the model. The model parameters were set with the following considerations: the number of trees in the forest was set to 1000, and the maximum depth of each tree was limited to prevent overfitting. The feature importance was evaluated using the Gini index and permutation importance. The random forest algorithm was implemented using the scikit-learn library in Python. A p-value <0.05 was considered statistically significant.

## Results

### General data

The baseline data of 100 patients with CBI was analyzed to investigate the correlation between JVBG analysis and the severity of CBI, as well as its predictive role in the length of ICU stay (Table 1). In the severe CBI group (n = 50), consisting of 39 males and 11 females, and the exceptionally severe CBI group (n = 50), with 34 males and 16 females, gender distribution did not show a significant difference between the groups (t=0.812, *P*=0.368). The presence of cerebral trauma, cerebral hernia, cerebral infarction, cerebral artery stenosis, and death did not demonstrate statistically significant differences between the two groups. However, there was a significant association between intracranial hemorrhage and severity of CBI (χ^2^ = 7.042, *P*=0.008). The exceptionally severe CBI group had a higher proportion of intracranial hemorrhage compared to the severe CBI group. Furthermore, patients in the exceptionally severe CBI group had a poorer GOS and GCS compared to those in the severe CBI group, with p-values of less than 0.001 for both parameters. Notably, the Acute Physiology and Chronic Health Evaluation (APACHE) score was significantly higher in the exceptionally severe CBI group compared to the severe CBI group (t=815.5, *P*=0.003).

**Table 1:** Comparison of baseline data, liver and kidney function between HF patients and HF+HRS patients.

Parameter	Severe<br>(n = 50)	Exceptionally severe<br>(n = 50)	t/χ^2^	P-value
Gender (M/F)	39/11	34/16	0.812	0.368
Cerebral trauma (Y/N)	14/36	8/42	1.457	0.227
Cerebral hernia (Y/N)	1/49	1/49	0	1
Intracranial hemorrhage (Y/N)	23/27	37/13	7.042	0.008
Cerebral infarction (Y/N)	11/39	4/46	2.824	0.093
Cerebral artery stenosis (Y/N)	1/49	0/50	None	1
Death (Y/N)	1/49	4/46	0.842	0.359
GOS (1/2/3/4)	12/3/4/13/18	19/7/8/15/1	19.867	< 0.001
GCS (3/4/5/6/7/8)	0/0/0/16/5/29	9/6/35/0/0/0	100	< 0.001
Age	57±13.97	57.46±12.76	0.172	0.864
APACHE	16.4±5.46	20.04±6.32	815.5	0.003

### Jugular blood gas analysis at one day after admission

The JVBG analysis at one day after admission was compared between the severe CBI group (n = 50) and the exceptionally severe CBI group (n = 50) (Table 2). The jugular venous oxygen saturation (SjvO_2_) and jugular venous partial pressure of oxygen (PjO_2_) at one day post-admission were significantly lower in the exceptionally severe CBI group compared to the severe CBI group, with p-values of less than 0.001 for both parameters (t=7.977 and 7.383, respectively). Moreover, arteriovenous oxygen difference (AVDO_2_) at one day after admission was markedly higher in the exceptionally severe CBI group compared to the severe CBI group (t=389, *P *< 0.001), indicating a greater oxygen extraction in the exceptionally severe CBI patients. Additionally, the arteriovenous oxygen content difference (AVDL) at one day post-admission was statistically significantly different between the groups (t=3.338, *P*=0.001), suggesting variations in oxygen extraction and consumption.

**Table 2:** Comparison of baseline data, liver and kidney function between HF patients and HF+HRS patients.

Parameter	Severe (n=50)	Exceptionally severe	Statistics	P-value
SjvO_2_ 1d (%)	66.07±7.08	55.14±6.61	7.977	< 0.001
PjO_2_ 1d (mmHg)	60.65±5.88	50.21±8.09	7.383	< 0.001
BlA 1d (mmol/L)	2.59±1.08	3±1.14	980.5	0.063
Hemoglobin 1d (g/dL)	11.22±2.19	11.5±1.6	0.736	0.464
SaO_2_ 1d (%)	96.82±1.75	96.19±1.47	1560	0.033
PaO_2_ 1d (mmHg)	126.36±40.88	116.07±31.42	1450.5	0.168
PaCO_2_ 1d (mmHg)	38.72±6.35	39.6±6.48	0.689	0.493
AVDO_2_ 1d (mmHg)	459.88±142.62	629.84±121.74	389	< 0.001
AVDL 1d (mL/dL)	-0.06±0.37	-0.33±0.41	3.338	0.001

### Jugular blood gas analysis at two days after admission

Significant differences were observed in jugular venous oxygen saturation (SjvO_2_) and jugular venous partial pressure of oxygen (PjO_2_) at two days postadmission, with lower values in the exceptionally severe CBI group compared to the severe CBI group (t= 1696 and 1604, respectively; *P*=0.002 and 0.015, respectively) (Table 3). Moreover, arteriovenous oxygen difference (AVDO_2_) at two days after admission was also found to be significantly higher in the exceptionally severe CBI group compared to the severe CBI group (t=2.478, *P*= 0.015), indicating increased oxygen extraction in the exceptionally severe CBI patients at this time point. Additionally, the arteriovenous oxygen content difference (AVDL) at two days post-admission exhibited a significant difference between the groups (t=1865, *P* < 0.001), suggesting notable variations in oxygen consumption.

**Table 3:** Jugular blood gas analysis at two days after admission.

Parameter	Severe (n = 50)	Exceptionally severe	Statistics	P-value
SjvO_2_ 2d (%)	56±9.29	50.75±6.5	1696	0.002
PjO_2_ 2d (mmHg)	49.5±10.94	43.94±12.14	1604	0.015
BlA 2d (mmol/L)	2.19±0.72	2.52±1.03	1033.5	0.136
Hemoglobin 2d (g/dL)	10.72±2.21	10.79±1.79	1188.5	0.674
SaO_2_ 2d (%)	97.37±1.49	97.09±1.28	1392	0.329
PaO_2_ 2d (mmHg)	121.16±30.28	112.97±24.44	1452	0.165
PaCO_2_ 2d (mmHg)	37.5±6.4	38.6±4.82	0.971	0.334
AVDO_2_ 2d (mmHg)	589.94±172.74	668.27±141.92	2.478	0.015
AVDL 2d (mL/dL)	0±0.3	-0.29±0.33	1865	< 0.001

### Jugular blood gas analysis at three days after admission

The results revealed significant differences in jugular venous oxygen saturation (SjvO_2_) and jugular venous partial pressure of oxygen (PjO_2_) at three days post-admission, with both parameters being lower in the exceptionally severe CBI group compared to the severe CBI group (t=1800.5, *P* < 0.001 and t=4.431, *P* < 0.001, respectively) (Table 4). Additionally, arteriovenous oxygen difference (AVDO_2_) at three days after admission was significantly higher in the exceptionally severe CBI group compared to the severe CBI group (t=3.111, *P* = 0.002), indicating a greater oxygen extraction in the exceptionally severe CBI patients at this time point. Moreover, the arteriovenous oxygen content difference (AVDL) at three days post-admission exhibited a statistically significant difference between the groups (t=1740.5, *P* < 0.001), suggesting notable variations in oxygen consumption.

**Table 4:** Jugular blood gas analysis at three days after admission.

Parameter	Severe (n = 50)	Exceptionally severe (n = 50)	Statistics	P-value
SjvO_2_ 3d (%)	56.36±7.89	50.09±8.28	1800.5	< 0.001
PjO_2_ 3d (mmHg)	49.66±7.93	41.38±10.57	4.431	< 0.001
BlA 3d (mmol/L)	1.87±0.5	2.14±1.02	1137.5	0.439
Hemoglobin 3d (g/dL)	10.48±1.55	10.65±1.78	1196.5	0.715
SaO_2_ 3d (%)	96.94±1.49	96.63±1.83	1341	0.532
PaO_2_ 3d (mmHg)	110.58±27.41	107.59±24.74	1292	0.775
PaCO_2_ 3d (mmHg)	38.76±5.69	39.41±4.89	0.612	0.542
AVDO_2_ 3d (mmHg)	568.59±142.47	660.63±153.16	3.111	0.002
AVDL 3d (mL/dL)	0±0.29	-0.22±0.38	1740.5	< 0.001

### Jugular blood gas analysis at four days after admission

The results indicated that jugular venous oxygen saturation (SjvO_2_) and jugular venous partial pressure of oxygen (PjO_2_) at four days post-admission were significantly lower in the exceptionally severe CBI group compared to the severe CBI group (t=3.53, *P* < 0.001 and t=1648, *P* = 0.006, respectively) (Table 5). Notably, the arteriovenous oxygen content difference (AVDL) also presented a significant difference between the groups at this time point (t=1620, *P* = 0.01), suggesting distinct variations in oxygen consumption.

**Table 5:** Jugular blood gas analysis at four days after admission.

Parameter	Severe (n = 50)	Exceptionally severe (n = 50)	Statistics	P-value
SjvO_2_ 4d (%)	55.64±7.61	50.33±7.42	3.53	< 0.001
PjO_2_ 4d (mmHg)	47.23±8.51	41.86±9.57	1648	0.006
BlA 4d (mmol/L)	1.87±0.59	2.08±0.69	984	0.067
Hemoglobin 4d (g/dL)	10.68±1.73	10.26±2.06	1335.5	0.558
SaO_2 _4d (%)	97.08±1.3	96.35±1.9	1528	0.056
PaO2 4d (mmHg)	108.92±25.8	103.08±24.69	1408.5	0.276
PaCO_2_ 4d (mmHg)	38.48±5.52	39.28±4.08	1223.5	0.857
AVDO_2_ 4d (mmHg)	591.66±134.06	628.99±143.06	1.346	0.181
AVDL 4d (mL/dL)	-0.04±0.27	-0.21±0.37	1620	0.01

### Jugular blood gas analysis at five days after admission

The results demonstrated that jugular venous oxygen saturation (SjvO_2_) and jugular venous partial pressure of oxygen (PjO_2_) at five days post-admission were significantly lower in the exceptionally severe CBI group compared to the severe CBI group (t=1631, *P* = 0.009 and t=1580, *P* = 0.023, respectively) (Table 6). Additionally, the arteriovenous oxygen content difference (AVDL) at five days after admission exhibited a statistically significant difference between the groups (t=1644, *P* = 0.006), indicating noticeable variations in oxygen consumption. Furthermore, arterial oxygen saturation (SaO_2_) at five days post-admission also presented a significant difference, with lower values in the exceptionally severe CBI group compared to the severe CBI group (t=1609.5, *P* = 0.013).

**Table 6:** Jugular blood gas analysis at five days after admission.

Parameter	Severe (n = 50)	Exceptionally severe (n = 50)	Statistics	P-value
SjvO_2_ 5d (%)	53.77±7.26	49.81±7.5	1631	0.009
PjO_2_ 5d (mmHg)	43.13±9.95	38.17±10.86	1580	0.023
BlA 5d (mmol/L)	1.78±0.46	2.14±0.76	887.5	0.012
Hemoglobin 5d (g/dL)	10.38±1.42	10.12±1.47	1402	0.296
SaO_2_ 5d (%)	97.43±1.56	96.34±2.3	1609.5	0.013
PaO_2_ 5d (mmHg)	110.29±23.05	106.64±26.05	1375	0.391
PaCO_2_ 5d (mmHg)	39.43±4.51	38.61±4.15	0.95	0.344
AVDO_2_ 5d (mmHg)	610.14±144.24	626.68±123.58	0.616	0.539
AVDL 5d (mL/dL)	-0.04±0.29	-0.2±0.3	1644	0.006

### The relationship between internal jugular venous blood gas analysis and the severity of craniocerebral injury

The relationship between internal JVBG analysis parameters and the severity of craniocerebral injury was investigated, revealing significant correlations (Table 7). Jugular venous oxygen saturation (SjvO_2_) and jugular venous partial pressure of oxygen (PjO_2_) at various time points post-admission demonstrated negative correlations with the severity of craniocerebral injury, with coefficients ranging from -0.236 to -0.627 and coefficients of determination (R2) ranging from 0.056 to 0.394 (*P* < 0.001 for all). Additionally, arteriovenous oxygen content difference (AVDO_2_) at multiple time points revealed positive correlations with craniocerebral injury severity, with coefficients ranging from 0.3 to 0.543 and R2 values ranging from 0.09 to 0.295 (*P* < 0.001 for all). Furthermore, the arteriovenous oxygen content difference (AVDL) at different time points also showed negative correlations with craniocerebral injury severity, with coefficients ranging from -0.32 to -0.412 and R2 values ranging from 0.102 to 0.17 (*P* < 0.001 for all). Additionally, variables such as arterial oxygen saturation (SaO_2_) and blood lactate (BlA) at specific time points demonstrated correlations with the severity of craniocerebral injury (*P* < 0.05). These findings underscore the potential of internal JVBG analysis parameters as predictors of craniocerebral injury severity, providing valuable insight into the predictive role of these parameters in understanding the trajectory of CBI.

**Table 7:** The relationship between internal jugular venous blood gas analysis and the severity of craniocerebral injury.

Parameter	r	R2	P value	Parameter	r	R2	P value
SjvO_2_ 1d	-0.627	0.394	< 0.001	AVDL 3d	-0.314	0.099	0.001
PjO_2_ 1d	-0.598	0.357	< 0.001	SjvO2 4d	-0.336	0.113	< 0.001
AVDO_2_ 1d	0.543	0.295	< 0.001	PjO2 4d	-0.287	0.082	0.004
AVDL 1d	-0.32	0.102	0.001	SaO2 4d	-0.221	0.049	0.027
SjvO_2_ 2d	-0.314	0.099	0.001	AVDL 4d	-0.242	0.059	0.015
PjO_2_ 2d	-0.236	0.056	0.018	SjvO2 5d	-0.261	0.068	0.009
AVDO_2_ 2d	0.243	0.059	0.015	PjO2 5d	-0.234	0.055	0.019
AVDL 2d	-0.412	0.17	< 0.001	BlA 5d	0.281	0.079	0.005
SjvO_2_ 3d	-0.364	0.133	< 0.001	SaO2 5d	-0.27	0.073	0.007
PjO_2_ 3d	-0.409	0.167	< 0.001	AVDL5d	-0.264	0.07	0.008
AVDO_2_ 3d	0.3	0.09	0.002				

### The relationship between internal jugular venous blood gas analysis and the ICU stays

Correlation analyses revealed several significant findings related to internal JVBG analysis and the length of ICU stay for CBI patients (Table 8). Notably, age demonstrated a statistically significant negative correlation with a *P* value of 0.025 and an R2 of 0.05, indicating a potential predictive role in the duration of ICU stay. SaO_2_ 1d and PaCO_2_ 1d also exhibited significant correlations with the length of ICU stay. Conversely, parameters such as gender, cerebral hernia, cerebral infarction, and APACHE showed non-significant associations. These findings suggest that specific internal JVBG analysis parameters may hold predictive value for the length of ICU stay in CBI patients, thus warranting further investigation and potential clinical implications.

**Table 8:** The relationship between internal jugular venous blood gas analysis and the ICU stay.

Parameter	r	R2	P value	Parameter	r	R2	*P* value
Gender	0.107	0.011	0.29	SjvO2 3d	0.078	0.006	0.441
Age	-0.224	0.05	0.025	PjO2 3d	0.076	0.006	0.453
Cerebral trauma	-0.061	0.004	0.549	BlA 3d	-0.139	0.019	0.168
Cerebral hernia	0.023	0.001	0.819	hemoglobin 3d	-0.018	0	0.861
Intracranial hemorrhage	0.157	0.025	0.118	SaO2 3d	-0.169	0.029	0.092
Cerebral infarction	-0.187	0.035	0.062	Pao2 3d	-0.063	0.004	0.536
Cerebral artery stenosis	0.118	0.014	0.242	PaCO2 3d	0.008	0	0.941
APACHE	0.009	0	0.928	AVDO2 3d	-0.11	0.012	0.276
Death	-0.102	0.01	0.311	AVDL 3d	-0.101	0.01	0.32
GOS	0.059	0.004	0.557	SjvO2 4d	-0.058	0.003	0.566

### Construction of multiple prediction model

**Figure 2:**
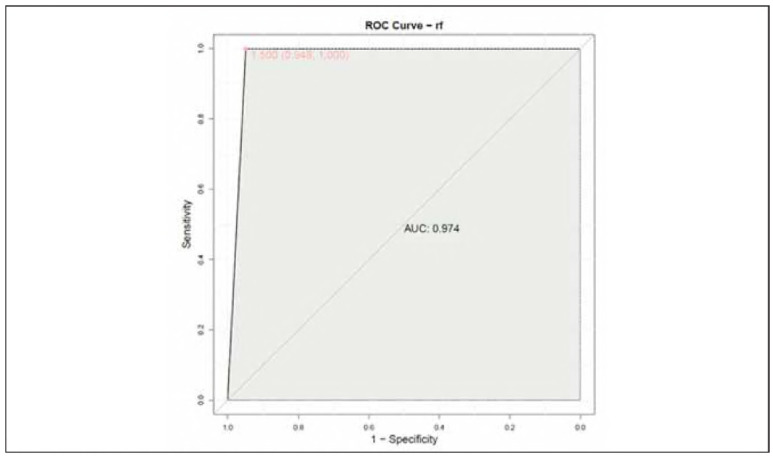
Multivariate predictive models for age, SaO_2_ 1d, and PaCO_2_ 1d.

To predict whether the length of hospitalization was greater than 14 days, age, SaO_2_ 1d and PaCO_2_ 1d were selected to construct a multivariate prediction model based on random forest algorithm (Figure 2). The AUC could reach 0.974, the sensitivity was 100%, and the specificity was 94.8%.

## Discussion

The results of this study provide substantial insights into the correlation between jugular venous blood gas (JVBG) analysis and the severity of CBI, as well as its predictive role in the length of ICU stay. CBI remains a significant public health concern, with profound implications for the affected individuals, their families, and healthcare systems globally [24] [25] [26]. The multifaceted impact of CBI, encompassing physical, cognitive, emotional, and functional domains, necessitates a comprehensive understanding of the factors influencing its severity, prognosis, and management [27] [28]. The use of JVBG analysis as a promising tool for assessing CBI severity and predicting patient outcomes has garnered increasing attention due to its direct reflection of cerebral metabolism and oxygenation [29] [30]. The findings of this study underscore the potential of JVBG parameters, including jugular venous oxygen saturation (SjvO_2_), jugular venous partial pressure of oxygen (PjO_2_), arteriovenous oxygen difference (AVDO_2_), and arteriovenous oxygen content difference (AVDL), as valuable indicators of CBI severity and predictive markers for the length of ICU stay.

The results of this study revealed several key findings that contribute to the body of knowledge regarding the use of JVBG analysis in the assessment and management of severe CBI. Firstly, the baseline data analysis showed that the presence of intracranial hemorrhage was significantly associated with the severity of CBI. This was consistent with existing literature, as intracranial hemorrhage was a known indicator of severe CBI and was associated with poor outcomes.

Moreover, the comparison of JVBG parameters between the severe CBI group and the exceptionally severe CBI group at different time points post-admission revealed significant differences. Specifically, jugular venous oxygen saturation (SjvO_2_) and jugular venous partial pressure of oxygen (PjO_2_) were consistently lower in the exceptionally severe CBI group, indicating compromised cerebral oxygenation in these patients. Our study found that patients with more severe CBI had significantly reduced SjvO_2_ and PjO_2_. These parameters are critical indicators of cerebral oxygenation. When SjvO_2_ and PjO_2_ are low, it reflects that the brain tissue may be experiencing hypoxia. This is not merely a numerical decrease but a warning sign of potential neurological deterioration. In clinical practice, identifying patients with low SjvO_2_ and PjO_2_ allows healthcare providers to take timely interventions, such as optimizing cerebral perfusion pressure, adjusting ventilation settings, or initiating neuroprotective strategies, which may help improve neurological outcomes and prevent further brain damage. Additionally, arteriovenous oxygen difference (AVDO_2_) was significantly higher in the exceptionally severe CBI group at multiple time points, suggesting increased oxygen extraction and metabolic demand in this cohort. These findings align with the pathophysiology of severe CBI, where compromised cerebral oxygenation and increased oxygen extraction reflect the severity of brain injury and the associated physiological stress.

The correlations between JVBG parameters and the severity of craniocerebral injury revealed significant relationships, further supporting the potential utility of JVBG analysis as a prognostic tool for CBI severity. Negative correlations between SjvO_2_, PjO_2_, and severity of craniocerebral injury underscore the association between impaired cerebral oxygenation and the severity of CBI. Conversely, positive correlations were observed between AVDO_2_ and the severity of craniocerebral injury, indicating higher oxygen extraction in patients with more severe CBI. These correlations highlight the physiological implications of JVBG parameters in reflecting the severity of brain injury and suggest their value as objective markers for CBI severity assessment.

Furthermore, the relationship between JVBG analysis and the length of ICU stay demonstrated that specific JVBG parameters, such as arterial oxygen saturation (SaO_2_) and partial pressure of carbon dioxide (PaCO_2_) at one day after admission, exhibited significant correlations with the length of ICU stay. These findings suggest that early assessment of JVBG parameters may aid in predicting the trajectory of CBI patients' clinical course and resource utilization, providing potential implications for individualized patient management and resource allocation.

The construction of a multiple prediction model based on age, SaO_2_ at one day after admission, and PaCO_2_ at one day after admission using the random forest algorithm further emphasized the potential of JVBG parameters in predicting the length of hospitalization. The high AUC value and sensitivity of the predictive model indicate the robust predictive capability of these JVBG parameters, offering promising prospects for their integration into clinical decision-making for CBI patients.

The results of this study have several clinical implications. Firstly, the significant correlations between JVBG parameters and the severity of CBI underscore the potential value of JVBG analysis as a complementary tool in CBI assessment, especially in cases of severe or exceptionally severe CBI. Early monitoring of JVBG parameters may provide valuable information regarding cerebral oxygenation and metabolic status, guiding clinical decision-making and potentially facilitating timely interventions to optimize cerebral perfusion and oxygenation in critically ill CBI patients. Additionally, the predictive role of JVBG parameters in determining the length of ICU stay has significant implications for resource allocation and healthcare management. Early identification of CBI patients at higher risk of prolonged ICU stay based on JVBG parameters could facilitate proactive resource allocation, individualized care planning, and timely escalation or de-escalation of care according to the patient's physiological status. This targeted approach may optimize resource utilization, improve patient outcomes, and enhance the overall efficiency of CBI management in the ICU setting. Furthermore, the findings of this study highlight the potential for JVBG parameters to serve as objective markers for CBI severity and prognosis, offering valuable insights into the physiological status of the injured brain. Integration of JVBG analysis into the routine assessment and monitoring of CBI patients may enhance the precision and granularity of patient care, allowing for tailored therapeutic interventions and prognostication based on real-time physiological data. Moreover, it can be combined with other clinical judgment tools to provide patients with a more comprehensive and accurate assessment. For instance, JVBG analysis can be integrated with neuroimaging techniques such as magnetic resonance imaging (MRI) and computed tomography (CT), which offer detailed information on the structure and anatomy of brain injuries. This integration enables clinicians to correlate the brain metabolic and oxygenation status indicated by JVBG parameters with structural findings from imaging studies. This comprehensive approach enhances the understanding of the extent of injury and its functional impact. In addition, JVBG analysis can be combined with the assessment of the recovery of consciousness (such as GCS and GOS). By monitoring the changes in JVBG parameters and combining them with the improvement or decline in the level of consciousness, clinicians can gain a more comprehensive understanding of the patient's neurological status and prognosis. This multimodal approach helps to formulate treatment strategies more wisely and predict the patient's prognosis more accurately.

Despite the valuable insights provided by this study, several limitations should be acknowledged. For instance, JVBG parameters can be influenced by various factors such as the patient's hemodynamic status, the presence of severe cardiopulmonary disease, and the accuracy of the blood sampling technique. These factors may affect the reliability and interpretation of JVBG results. Additionally, while JVBG provides valuable information about cerebral oxygenation and metabolism, it does not account for other potential factors that may influence the severity of CBI and ICU stay duration, such as the extent of brain edema, intracranial pressure, the presence of secondary brain injuries, or the effectiveness of treatment interventions. Other factors like patient age, pre-existing medical conditions, genetic predispositions, and the timeliness of medical intervention can also significantly impact the severity of CBI and ICU stay duration. The retrospective nature of the study and the use of data from a single center may limit the generalizability of the findings to diverse patient populations and healthcare settings. Additionally, the small sample size and the focus on specific JVBG parameters warrant further validation and exploration of a broader range of physiological markers to comprehensively assess the utility of JVBG analysis in CBI management.

## Dodatak

### Acknowledgements

The authors are grateful to all participants in the present study.

### Funding

The authors disclosed receipt of the following financial support for the research, authorship, and publication of this article: This work was supported by Health Research Project of Anhui Province in 2022 (grant number AHWJ2022c033).

### Statement of patient consent

The study was approved by the Ethics Committee of Bozhou People's Hospital. Informed consent was waived for this retrospective study due to the exclusive use of de-identified patient data, which posed no potential harm or impact on patient care. This waiver was approved by the Ethic Committee of Bozhou People's Hospital Institutional in accordance with regulatory and ethical guidelines pertaining to retrospective research studies.

### Author contributions

All authors contributed to the study conception and design. Material preparation, data collection and analysis were performed by Bin Qi, Wenjie Du, He Zhang, Yuhui Jiang and Ming Zhuo. The first draft of the manuscript was written by Bin Qi and Wenjie Du and Haixia Xu commented on previous versions of the manuscript. All authors read and approved the final manuscript.

### Data availability statement

All data generated or analyzed in this study are included in the present manuscript.

### Conflict of interest statement

All the authors declare that they have no conflict of interest in this work.
